# A Systematic Review and Meta-Analysis of Burnout Among Healthcare Workers During COVID-19

**DOI:** 10.3389/fpsyt.2021.758849

**Published:** 2021-11-10

**Authors:** Sulmaz Ghahramani, Kamran Bagheri Lankarani, Mohammad Yousefi, Keyvan Heydari, Saeed Shahabi, Sajjad Azmand

**Affiliations:** ^1^Health Policy Research Center, Institute of Health, Shiraz University of Medical Sciences, Shiraz, Iran; ^2^Department of Medicine, School of Medicine, Semnan University of Medical Sciences, Semnan, Iran; ^3^Student Research Committee, School of Medicine, Mazandaran University of Medical Sciences, Sari, Iran; ^4^Gastrointestinal Cancer Research Center, Non-Communicable Diseases Institute, Mazandaran University of Medical Sciences, Sari, Iran; ^5^Medical Ethics and Philosophy of Health Department, Shiraz Faculty of Medicine, Shiraz University of Medical Sciences, Shiraz, Iran

**Keywords:** burnout, healthcare workers, COVID-19, systematic review, meta-analysis

## Abstract

Burnout among healthcare personnel has been exacerbated by the COVID-19 pandemic's unique features. During the COVID-19 pandemic, this systematic review and meta-analysis aims to provide a complete assessment of the prevalence of burnout across various healthcare personnel. Until January 2021, systematic searches for English language papers were conducted using PubMed, Scopus, EMBASE, Web of Science, Cochrane Library, and ProQuest. Thirty observational studies were found after conducting systematic searches. The pooled overall prevalence of burnout was 52% [95% confidence interval (CI) 40–63%]. Pooled emotional exhaustion (EE), depersonalization (DP), and lack of personal accomplishment (PA) were 51% (95% CI 42–61%), 52% (95% CI 39–65%), and 28% (95% CI 25–31%), respectively. This study demonstrated that nearly half of the healthcare workers experienced burnout during the COVID-19 pandemic. In the studies that were included, non-frontline COVID-19 exposed healthcare personnel also experienced burnout. From high to lower middle-income countries, there was a gradient in the prevalence of total burnout, EE, and lack of PA. Further studies on burnout in low and lower-middle-income countries are suggested. A uniform diagnostic tool for the assessment of burnout is warranted.

## Introduction

The coronavirus disease 2019 (COVID-19) pandemic has affected various aspects of communities, including political, economic, social, psychological, and health management elements as well as their physical health (1–4). The physical and emotional well-being of healthcare professionals plays a major contribution in pandemic containment. As evidenced during previous outbreaks, such as severe acute respiratory syndrome (SARS) and Middle East Respiratory Syndrome (MERS), the psychological well-being of healthcare professionals is of crucial importance for health authorities, particularly their burnout (5–7). Burnout is a three-dimensional affective response to continuous work-related stress and is common in workplaces where employees spend more time supporting others. Both individual characteristics of healthcare workers and work-related factors contribute to this situation (5, 8–12). Burnout includes emotional exhaustion (EE), depersonalization (DP), and a loss of personal accomplishment [PA] (13, 14). EE occurs when employees feel tired or having little energy to participate emotionally. DP contains developing negative attitudes and feelings toward others who perform labor for them. Those who experience reduced PA tend to underestimate their abilities to carry out tasks and interact with others. In the wake of the COVID-19 pandemic, research into burnout among healthcare workers has evolved. There is evidence indicating the negative effects of burnout on the number of healthcare workers, which depends on several factors such as patient-facing roles [doctors, nurses, and other clinical] (15, 16), frontline exposure with COVID-19 patients (17), and country income level (16, 18). In addition, burnout has negative repercussions for healthcare staff as well as patients (19–25).

Some burnout reviews have focused on particular populations and/or groups. Nevertheless, according to the best knowledge of the author, no meta-analysis has been conducted on the overall prevalence of burnout among healthcare workers. Most of the currently published studies have focused on burnout among healthcare staff of COVID-19 wards (26), physicians (27), or female healthcare workers (28), and primarily described the triggers and risk factors, as well as interventions and suggestions for burnout reduction. One study summarized the prevalence of nurse burnout during pandemics (29). Nonetheless, a thorough assessment and meta-analysis of the prevalence of burnout among all healthcare workers during the COVID-19 epidemic appears desirable. Understanding the prevalence of burnout and the characteristics of high-risk groups would provide useful evidence for health policymakers to better develop screening procedures intended to identify vulnerable healthcare professionals as well as implementing appropriate pro-active holistic measures without delay (30, 31). The goal of this systematic review and meta-analysis was to present a comprehensive picture of the prevalence of burnout among healthcare workers during the COVID-19 pandemic.

## Methods

### Study Design

For conducting this systematic review and meta-analysis, the Cochrane criteria and Preferred Reporting Items for Systematic Reviews and Meta-Analysis (PRISMA) recommendations were utilized. The protocol of this systematic review and meta-analysis has been registered (code: CRD42021229152) in the international prospective register of systematic reviews (PROSPERO).

### Search Strategy and Data Sources

By January 1, 2021, a systematic search of peer-reviewed and English-language materials relating to the study question, “What is the prevalence of burnout among healthcare providers in the face of COVID-19?,” has been completed. First, a rapid and initial search of the Scopus, Cochrane Library database, and PROSPERO databases was performed to ensure that no registered systematic reviews precisely matched the purpose of the current investigation. There were no related articles found. In the next step, PubMed, Scopus, EMBASE, Science Direct Web of Science, Cochrane Library, and ProQuest were all searched. Gray literature, which included Internet sites, conference papers, and dissertations, was also searched. Also, the research team tried to obtain any relevant unpublished studies through searching of registries such as clinicaltrials.gov. The “AND” operator was used to perform a search between groupings of words regarded to represent a different understanding. Between the synonyms, the “OR” operator was also utilized. The search was conducted in the article's “Title, Abstract, and Keyword” sections. Besides the MeSH and Emtree thesauruses, the free-text method was also employed to achieved appropriate terms. Three compartments of PICO (population, intervention, comparison, and outcome), including population, intervention, and outcome, were considered in the search strategy process. Initially, the search string was created for the PubMed database and then adapted for other interested databases. Supplementary File 1 shows the search strings that were used for the four main databases.

### Study Selection

For each article, a thorough list of references was compiled. First, two authors assessed the titles of all articles in the database (SGH, SA). Articles that matched the inclusion criteria and were about the research objective were chosen. The abstracts of the selected papers were then read by the two authors in the following phase.

Articles about the prevalence of burnout in healthcare workers during the COVID-19 pandemic were chosen. All of the above steps were repeated twice. In the event of a disagreement regarding whether or not to include the study, the senior author (KBL) was the final evaluator. The papers contained the prevalence of burnout and/or three burnout dimensions (EE, DP, and the lack of PA) within the article or its supporting information considered acceptable for meta-analysis.

### Inclusion Criteria

According to the PICO compartments, P denotes the study population, I represents COVID-19 disorders, and O represents burnout. C, or the comparison group, was not examined in this investigation because there was no comparison group. Burnout is defined per each study's goal. Articles in English were included. Unpublished articles (Gray literature), instructions, guidelines, and reports from recognized organizations, were also reviewed. Articles should be related to the research question and should be based on a valid and reliable study tool. Only articles that had been peer-reviewed were chosen. Letters and short communications which have the required data were included in the study. Original articles, including cross-sectional, case-control, and cohort studies, were included.

### Exclusion Criteria

Articles with no factors related to the research topic (prevalence of burnout) and articles examining the burnout of medical students, residents, and other health-related students were omitted. Furthermore, reports revealed burnout of specific wards or experts (other than intensive care unit specialists and infectious specialists who may have direct contact with COVID-19 patients), studies that assessed burnout with a single item, and studies in which only evaluated emotional exhaustion were omitted. In addition, case reports, reviews, protocols, editorials, and qualitative studies were not included.

### Quality Appraisal

The final included full texts' quality was assessed using 22 items from the Strengthening the Reporting of Observational Studies in Epidemiology (STROBE) checklist. This checklist includes questions for each of the following sections: title and abstract, introduction, methods, results, discussion, and other information.

A description of the study design, setting, participants and variables, data sources/measurement, bias, study size, quantitative variables, and statistical procedures were among the methodological criteria.

For all items, “1” indicates the presence of the item, “0” shows the absence of the item, and “?” indicates that the criteria were not met completely or were not applicable.“0,” “?,” and “1” will be rated as 0, 1, and 2, respectively, in the computation of total study quality. The sum of the scores for each study's quality will next be computed. The quality of each study was graded as either good (most criteria met with a low risk of bias, score 39–44), fair (some criteria met with a moderate risk of bias, score 33–38), or poor (i.e., few criteria met and with a high risk of bias, score <33). The two authors (SA, MY) assessed quality independently, and disagreements were resolved by consensus or by consulting a third senior researcher (KBL). Low-quality studies will be included among the other research qualities. If meta-analysis is possible, the impact of these low-quality studies on the pooled effect will be examined using subgroup analysis and sensitivity analysis. We did not exclude them from the final analysis.

### Data Extraction

Following a thorough reading of the articles, the necessary information was retrieved using the summary and collection form. The title, responsible author, the sample size of the study, country and time of the study, study design, study participants based on their patient-facing roles (doctor, nurse, and other clinical), exposure of the participants to COVID-19 patients in the workplace, diagnostic instrument, and findings were all provided on this form. For each of the selected articles, summary forms were filled.

### Data Analysis

All meta-analyses were carried out using the Metaprop tool in STATA 11.0. (Stata Corp., College Station, TX, USA), and the exact binomial approach was used to obtain the 95% confidence interval. To assess heterogeneity, *I*-square (*I*^2^) was used. Due to the heterogeneity of the included studies, a random-effects meta-analysis was used to pool the prevalence (*I*^2^ ≥ 50%). To identify the cause of heterogeneity, subgroup analyses were performed based on the moderator's factors [country income level, study participants based on their patient-facing roles (doctor, nurse, and other clinical), exposure of the participants to COVID-19 patients in the workplace, sample size, time of data collection, and diagnostic instrument].

Income levels of countries are collected from the World Bank's most recent updates: https://data.worldbank.org and classified as low income (LIC), high income (HIC), lower middle income (LMIC), and upper middle income (UMIC).

Participants in the study, based on their patient-facing roles, were divided into two groups: nurses and/or physicians and mixed healthcare workers.

Based on the exposure of the participant to COVID-19 patients in the workplace, three categorizations were developed: yes (only participants with exposure to COVID-19 cases were studied), no (participants were not exposed to COVID-19 patients), and mixed exposure (both groups were studied) were developed.

Nurses and/or physicians include doctors or nurses, nurses and physicians, MDs and specialists, medical doctors (MDs) and nurses, MDs, and nurses.

Based on the date stated for the end of the data collection period, the time of data collection was categorized as the first 3 months of the pandemic (January, February, and March), and the following months (April, May, June, July, and after that).

Lists of diagnostic tools were 1—versions of MBI including Maslach Burnout Inventory-Human Services Survey for Medical Personnel [MBI-HSS (MP)], Maslach Burnout Inventory Human Services Survey (MBI-HSS), and Maslach Burnout Inventory-General Survey (MBI-GS); 2—versions of MBI modified or adapted (Chinese, Spanish, etc.); and 3—other tools (Copenhagen Burnout Inventory, the Stanford Professional Fulfillment Index, the Professional Quality of Life Questionnaire, and the Oldenburg Burnout Inventory).

Four of the seven studies that used adapted or modified MBI versions used a language-specific adapted version, including three Chinese (8, 32, 33) questionnaires and one Spanish (34) questionnaire. Out of the three Chinese adapted versions, two studies used a reliable and valid 15-item Chinese version of the MBI (8, 32) and one used a 22-item Chinese version of the MBI-HSS [MP] (33). In addition, the MBI-HSS Spanish adaption (34) includes a valid and reliable 22-item questionnaire. Three studies (35–37) utilized abridged versions of MBI. Table 1 contains information on the diagnostic instruments utilized in various studies.

**Table 1:** Characteristics of 30 studies included in systematic review.

**References**	**Month of data collection^I^**	**Country income level^II^**	**Study type**	**Frontline yes/no/mixed exposure^III^**	**Study participants^IV^**	**Age^V^**	**Sample size**	**Diagnostic tool (item used for meta-analysis)**
Abdelghani et al. (38)	Later months	Egypt, LMIC	Cross-sectional	Yes	Nurses and/or physicians	34.6 (6.04)	320	Maslach Burnout Inventory-Human Services Survey for Medical Personnel [MBI-HSS (MP)] high level for emotional exhaustion and depersonalization and low levels for personal accomplishment)^a^
Abdelhafiz et al. (39)	Later months	Egypt, LMIC	Cross-sectional	Mixed exposure	Nurses and/or physicians	33.42 (5.28)	220	Maslach Burnout Inventory-Human Services Survey (MBI-HSS) (moderate and high for emotional exhaustion and depersonalization were combined and low of personal accomplishment)^a^
Azoulay et al. (40)	Later months	85 countries, mixed	Cross-sectional	Yes	Nurses and/or physicians	45 (39–53)	848	Maslach Burnout Inventory (for overall burnout severe level, for emotional exhaustion and depersonalization moderate and severe were combined and for personal accomplishment low levels)^a^
Barello et al. (41)	Was not stated	Italy, HIC	Cross-sectional	Yes	Mixed healthcare workers	40 (11)	376	Maslach Burnout Inventory (high and moderate scores were combined for emotional exhaustion and depersonalization and low for personal gratification)^a^
Barello et al. (42)	Later months	Italy, HIC	Cross-sectional	Yes	Mixed healthcare workers	41 (11)	532	Maslach Burnout Inventory (only high level of Emotional exhaustion and depersonalization and low for personal accomplishment)^a^
Chen et al. (17)	First 3 months	China, UMIC	Cross-sectional	Mixed exposure	Mixed healthcare workers		902	15 Items Chinese version of Maslach Burnout Inventory^b^
Chen et al. (32)	Later months	China and Taiwan, UMIC	Cross-sectional	Mixed exposure	Nurses and/or physicians	33.1 (7.5)	12,596	Maslach Burnout Inventory-General Survey (moderate and high for emotional exhaustion and depersonalization were combined and high for lack of personal accomplishment)^a^
Di Monte et al. (43)	Later months	Italy, HIC	Cross-sectional	No	Nurses and/or physicians	55.13 (11.40)	102	Maslach Burnout Inventory, high and moderate combined for emotional exhaustion and depersonalization and low for personal accomplishment^a^
Dobson et al. (44)	Later months	Australia, HIC	Cross-sectional	Mixed exposure	Mixed healthcare workers	19–29: 75 (23.7%), 30–39: 100 (31.6%), 40–49: 69 (21.8%), 50 or over: 72 (22.8%)	320	Stanford Professional Fulfillment Index (symptoms of burnout)^c^
Duarte et al. (45)	Later months	Portugal, HIC	Cross-sectional	Mixed exposure	Mixed healthcare workers	38 (10)	2,008	Copenhagen Burnout Inventory (high levels for each of three different dimensions: personal burnout, work-related, and client-related burnout^c^
Elhadi et al. (36)	Later months	Libya, UMIC	Cross-sectional	No	Mixed healthcare workers	33.08 (7.25)	532	The English version of the Abbreviated Maslach Burnout Inventory (aMBI; for overall burnout presence of both emotional exhaustion and depersonalization, high level of emotional exhaustion and depersonalization, and low level of personal accomplishment)^b^
Evanoff et al. (46)	Later months	Washington University in St. Louis, HIC	Cross-sectional	Mixed exposure	Mixed healthcare workers		915	Professional Fulfillment Index (PFI; high overall burnout score >1.33)^c^
Giusti et al. (47)	Later months	Italy, HIC	Cross-sectional	Mixed exposure	Mixed healthcare workers	44.6 (13.5)	330	Maslach Burnout Inventory (high and moderate combined for emotional exhaustion and depersonalization and low for personal accomplishment)^a^
Gómez-Galán et al. (48)	Later months	USA, HIC	Cross-sectional	Yes^d^	Nurses and/or physicians			The Stanford Professional Fulfillment Index (SPFI; presence of burnout)^c^
Hu et al. (33)	First 3 months	China, UMIC	Cross-sectional	Yes	Nurses and/or physicians	30.99 (6.17)	2,014	22 Items Chinese version of the Maslach Burnout Inventory: Human Services Survey (MBI-HSS)for Medical Personnel (MP; moderate and high for emotional exhaustion, depersonalization were combined for n and low of personal accomplishment was reported for n)^b^
Kholmogorova et al. (49)	Later months	Russia, UMIC	Cross-sectional (not stated)	Yes	Mixed healthcare workers	36.1 (21–61)	120	Maslach Burnout Inventory (three dimensions were categorized as low, middle, and high level. Middle and high levels for emotional exhaustion, depersonalization, and high reduction of personal achievements were reported for n)^a^
Lázaro-Pérez et al. (50)	Later months	Spain, HIC	Descriptive study	Not clearly stated	Mixed healthcare workers	<41: 75 (47.8%) 41–60: 66 (42.0%) >60: 16 (10.2%)	157	Maslach and Jackson's scale (for Emotional Exhaustion and Depersonalization medium/high values and for personal accomplishment, low value were used for n)^a^
Liu et al. (8)	First 3 months	China, UMIC	Cross-sectional	Mixed exposure	Nurses and/or physicians	20–29: 198 30–39: 40 40–49: 191 >50: 85	880	15 Items Chinese version of the Maslach Burnout Inventory (CMBI): n were reported as emotional exhaustion, depersonalization, or reduced personal accomplishment. Overall burnout combined of mild burnout (only one of the three dimensions is positive), moderate burnout (arbitrary two of the three dimensions are positive), and severe burnout (all the three dimensions are positive) reported as %^b^
Luceño-Moreno et al. (34)	Later months	Spain, HIC	Cross-sectional	Yes	Mixed healthcare workers	43.88 (*SD* = 10.82, ranging between 19 and 68)	1,422	22 Items Spanish adaptation of the Maslach Burnout Inventory-MBI-HSS (moderate and high for emotional exhaustion and depersonalization were combined for n and low of personal accomplishment was reported for n)^b^
Martínez-López et al. (51)	Later months	Spain, HIC	Online survey	Yes	Mixed healthcare workers	Average: 41.8 <30: 35 (22.3%)31–40: 40 (25.5%)41–50: 30 (19.1%)51–60: 36 (22.9%)>60: 16 (10.2%)	157	Maslach Burnout inventory (medium and high for emotional exhaustion and depersonalization were combined and low personal accomplishment was reported)^a^
Matsuo et al. (52)	Later months	Japan, HIC	Cross-sectional	Yes	Mixed healthcare workers	30.5 (26–40)	312	Maslach Burnout Inventory (high levels of exhaustion (>3.5) plus either high cynicism (>3.5) or low professional efficacy (<2.5) were selected as the primary criteria for burnout)^a^
Miguel-Puga et al. (37)	Was not stated	Mexico, UMIC	Cross-sectional	Yes	Mixed healthcare workers	19–58 years old	204	The short version of the Burnout Measure by Malach–Pines and number of healthcare workers who had high score of burnout (score ≥3.5) reported in three separate evaluations but we only report the third (=the last) occasion^b^
Park et al. (53)	Later months	The Republic of Korea, HIC	Cross-sectional	Mixed exposure	Nurses and/or physicians	Median (IQR): 41 (37–48)	115	The Maslach Burnout Inventory-Human Services Survey (MBI-HSS): standardized thresholds set out in the MBI-HSS manual was applied for emotional exhaustion and depersonalization; lack of personal accomplishment was reported for n. Overall burnout was defined as a high score in either the emotional exhaustion or depersonalization subscale^a^
Ruiz-Fernández et al. (54)	Later months	Spain, HIC	Cross-sectional	Mixed exposure	Nurses and/or physicians	46.7 (10.2)	506	The Professional Quality of Life Questionnaire: percent of medium and high burn out were combined for %/number was not reported^c^
Sayilan et al. (55)	Later months	Turkey, UMIC	Cross-sectional	Yes	Nurses and/or physicians	28.03 (5.99)	267	The Maslach Burnout Inventory (three dimentions were categorized as low-moderate and high; moderate and high for emotional exhaustion and depersonalization (stated personalization in tables) were combined and low for personal accomplishment was reported for n)^a^
Roslan et al. (56)	Later months	Malaysia, UMIC	Cross-sectional (for prevalence of burnout)	Mixed exposure	Mixed healthcare workers	<40 years: 682 and 40 years and more than 40 years 211	893	The Malay-Translated Copenhagen Burnout Inventory (CBI)^c^
Tan et al. (57)	Later months	Singapore, HIC	Cross-sectional	Mixed exposure	Mixed healthcare workers	36.84 (9.95)	3,075	The Oldenburg Burnout Inventory (OLBI): burnout was determined with a cutoff of 2.25 for exhaustion and 2.10 for disengagement^c^
Khasne et al. (58)	Was not stated	India, LMIC	Prospective, cross-sectional	Mixed exposure	Mixed healthcare workers	21–30: 380, 31–40: 784, 41–50: 478, 51–60: 225, more than 61:129	2,026	The Copenhagen Burnout Inventory: personal burnout, work-related burnout, and client-related burnout namely pandemic-related burnout^c^
de Wit et al. (35)	Later months	Canada, HIC	Mixed-methods study(cohort and qualitative)	Not clearly stated	Nurses and/or physicians	Median (IQR): 41 (35–50)	468	The single item measures of emotional exhaustion and depersonalization from the Maslach Burnout Inventory which have been shown to correlate to the emotional exhaustion and depersonalization domains from the Maslach Burnout Inventory^b^
Zhang et al. (59)	First 3 months	China, UMIC	Prospective observational survey	Yes	Nurses and/or physicians	30.28 (5.49)	107	Maslach Burnout Inventory (three dimensions were categorized as mild-moderate and severe: moderate and severe for emotional exhaustion; depersonalization were combined and severe lack of personal accomplishment was reported for n)^a^

a *MBI*.

b *Adapted MBI*.

c *Other tools*.

d *Medical critical care physicians*.

I *Based on the date stated for termination of data collection: period were categorized as first 3 months of pandemic (January, February, and March of 2020), and afterwards, months*.

II *HIC: high income, LMIC: lower middle income, UMIC: upper middle income countries. Income level derived from the latest updates of World Bank: https://data.worldbank.org/*.

III *Participants in the study were divided into two groups: mixed healthcare workers, nurses and/or physicians*.

IV *Based on the exposure of the participant to COVID-19 patients in workplace, there are three categorizations: yes (only participants with exposure to COVID-19 cases were studied), no (participants were not exposed to COVID-19 patients), and mixed exposure (both groups were studies)*.

V *Age is presented differently: mean (SD), range, mean (range), median (interquartile), range: N, and range: N(%)*.

## Results

### Identification and Selection of Studies

**Figure 1:**
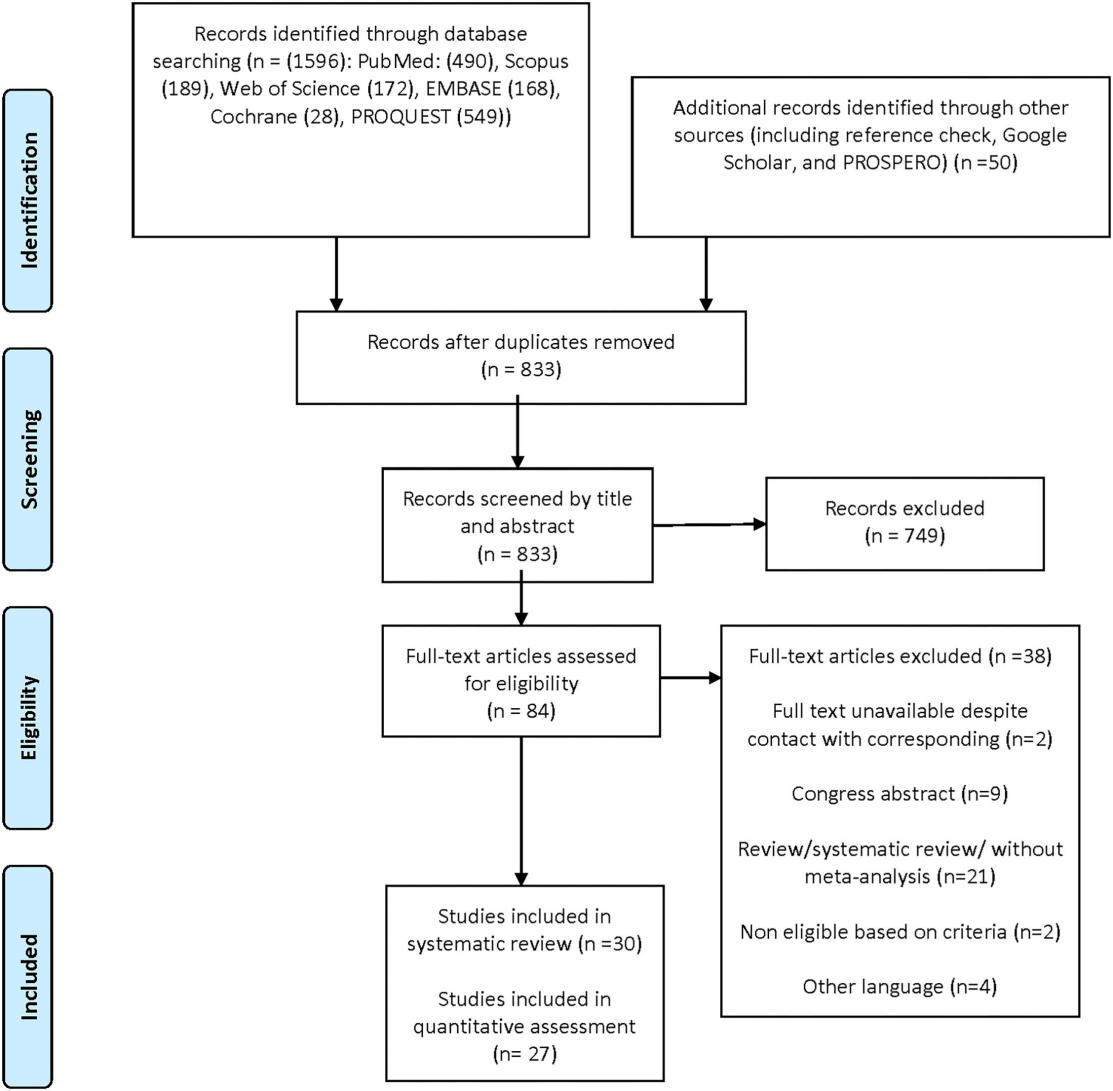
The flowchart of the literature searchaccording to PRISMA.

According to PRISMA principles, Figure 1 depicts the flowchart of the literature search. Through electronic databases, we first discovered 1,646 possible records, of which 833 remained after deleting duplicates. Seven hundred forty-nine records were eliminated after the titles and abstracts were screened. Finally, in this systematic review, we included 30 studies (8, 17, 32–59) of which 27 had sufficient data for the meta-analysis. Three studies neither reported the overall prevalence nor the three dimensions of burnout, due to distinctive study tools, so they were excluded from the meta-analysis (45, 56, 58).

### Characteristics of the Studies

Twenty-nine of the 30 observational studies included were cross-sectional. The study population included 32,724 healthcare workers. Three of the studies were not original research publications (41, 42, 52). The main characteristics of the 30 studies included in our systematic review are shown in Table 1.

Thirteen studies examined overall burnout using a variety of different study tools. Burnout dimensions such as EE and DP were pooled across 19 studies, whereas the lack of PA was pooled across 18 studies. Each of these studies used MBI, or one of its variants, in its entirety or in modified and adapted forms. There were no investigations into LICs, three into LMICs, while the remaining studies came from HICs and UMICs. Seventeen studies looked at the prevalence of burnout in mixed healthcare workers, whereas 13 looked at burnout in nurses, physicians, and nurses and physicians. The number of frontline vs. non-frontline participants, the timeframe when data collection ended, the age distribution, and the total sample size of the studies are all specified in Table 1.

### Quality Assessment

Supplementary Table 2 summarizes the quality assessments of the 27 original cross-sectional studies included in this study. Except for one study that was of fair quality (49), all of the others were of good quality, according to the STROBE checklist. The most systematic bias was failing to acknowledge the source of financing and failing to describe steps to address potential bias sources. Another common bias was that the eligibility criteria, as well as the sources and methods of participant selection, were not properly explained.

### Synthesized Findings

**Figure 2:**
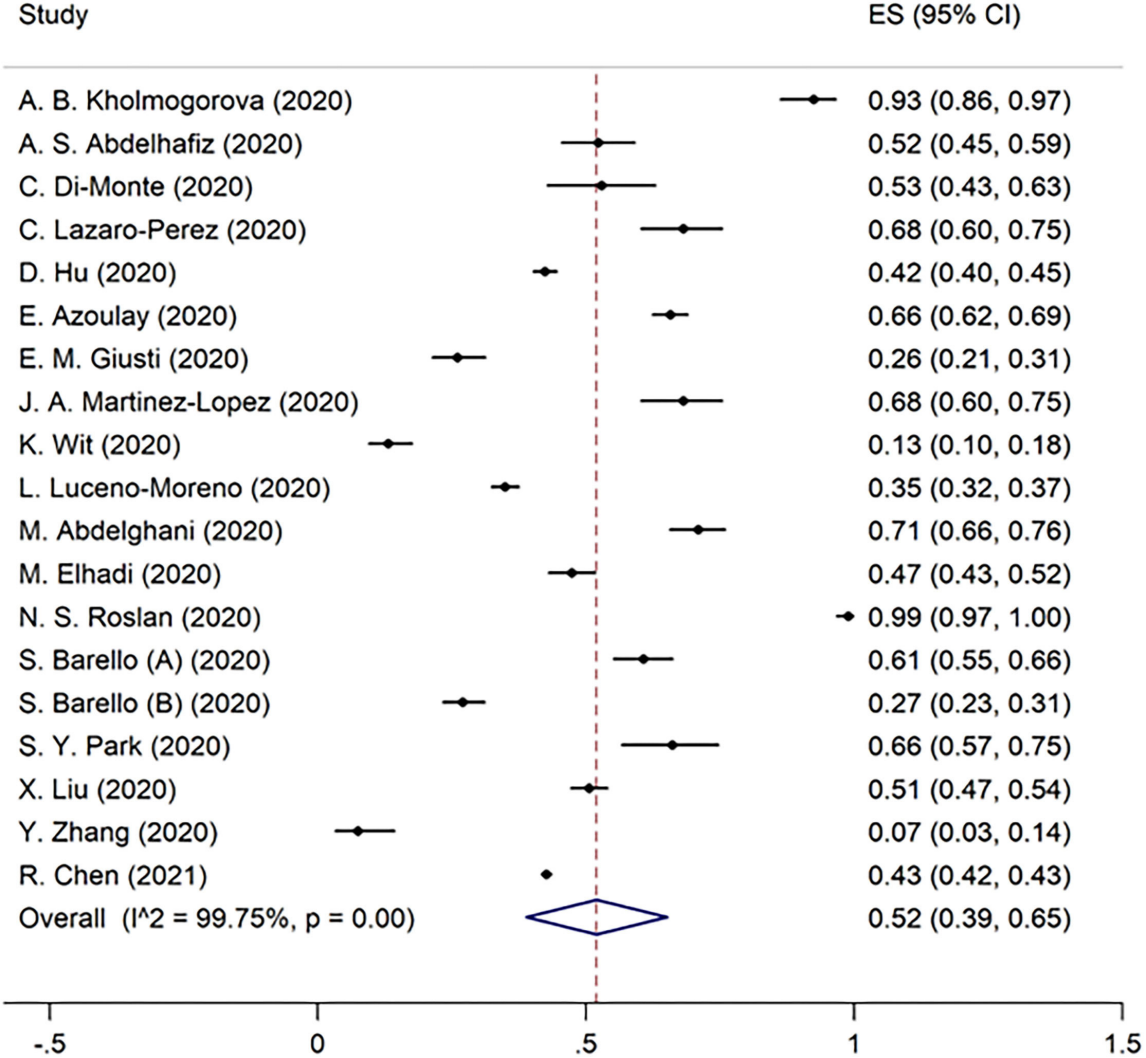
The pooled prevalence of overall burnout in included studies.

**Figure 3:**
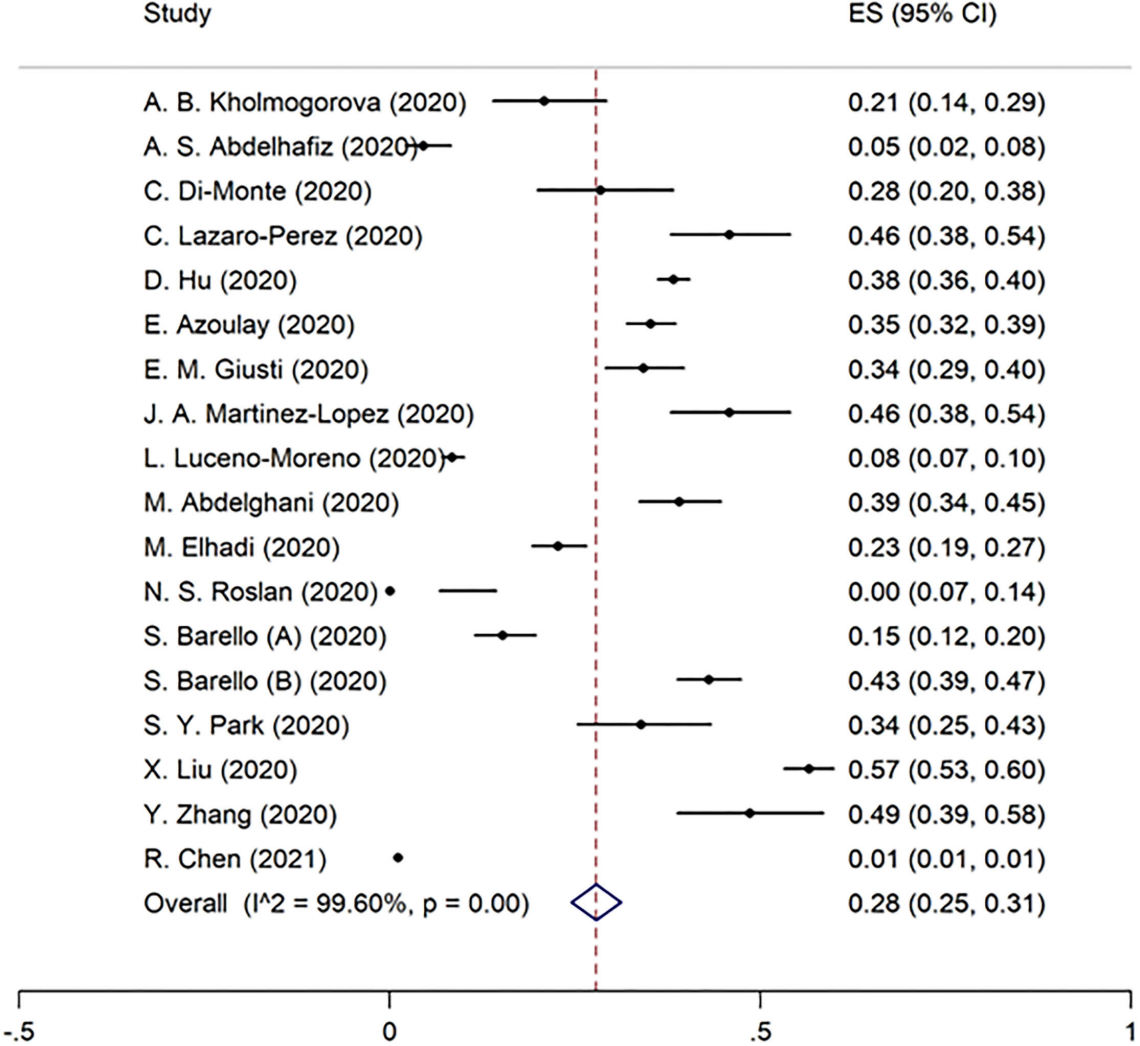
The pooled prevalence of emotional exhaustion in included studies.

**Figure 4:**
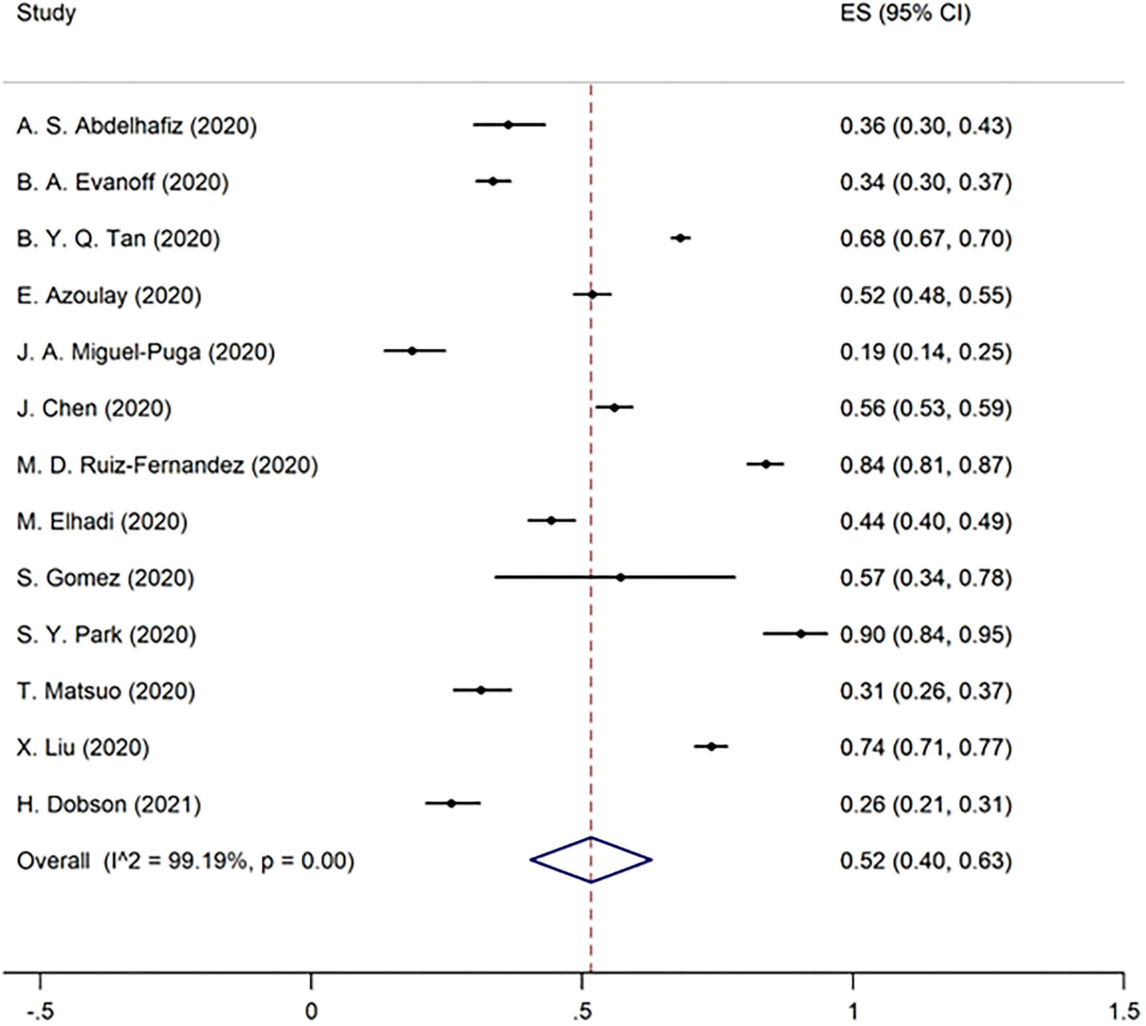
The pooled prevalence of depersonalization in included studies.

**Figure 5:**
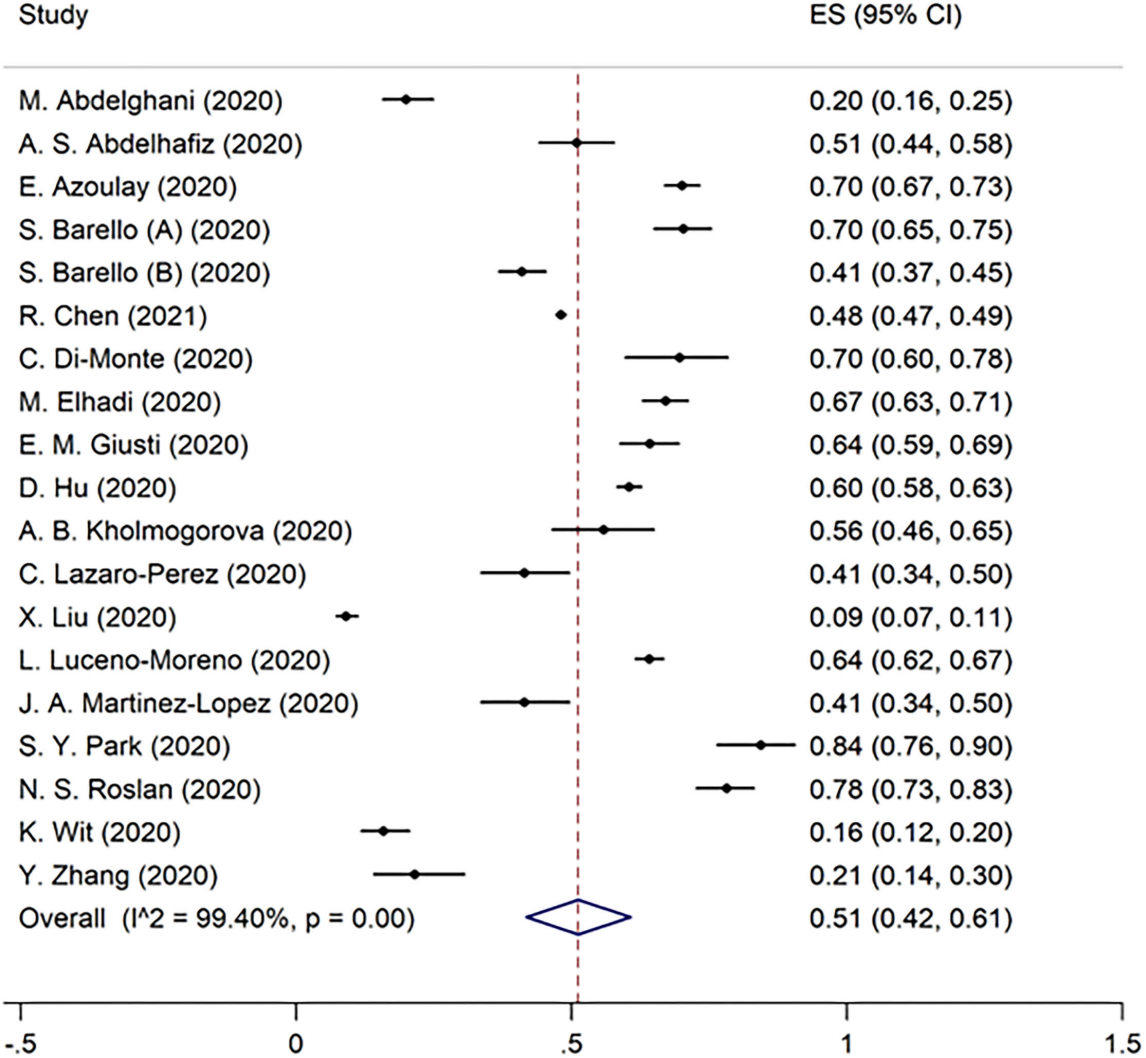
The pooled prevalence of lack of personal accomplishment in included studies.

Overall burnout in the included studies was 52% [95% CI 40–63%] (Figure 2). EE, DP, and the lack of PA were found to be 51% (95% CI 42–61%), 52% (95% CI 39–65%), and 28% (95% CI 25–31%) in the pooled data, respectively (Figures 3–5). Because the data on burnout scales was highly heterogeneous, subgroup analysis was used to determine the source of variation. The three non-original research publications had no significant impact on the overall results, according to sensitivity analysis.

### Subgroup Analysis

Overall burnout among the classified participants was highest among the physician and/or nurse groups at 66% (95% CI 51–81%). The mixed healthcare workers group, however, had the highest prevalence of EE and DP at 56% (95% CI 48–64%) and 53% (95% CI 37–69%), respectively. Moreover, the percentage of the mixed healthcare workers who did not have enough PA was the greatest at 29% (95% CI 18–40%).

Burnout was found to be high in studies that included both frontline and non-frontline participants (mixed exposure), with 55% (95% CI 40–69%) reporting burnout. In two studies, the non-frontline exposure group reported a high level of EE at 68% (95% CI 64–71%), while frontline exposure in 10 studies was associated with the highest level of DP at 57% (95% CI 35–78%) and a lack of PA at 29% (95% CI 17–41%).

Two studies that reported data collection termination for burnout during the first 3 months of the pandemic (January, February, and March) found a high overall burnout rate of 66% (95% CI 64–68%). In the following months, 15 studies found that the prevalence of EE and DP was 54% (95% CI 46–62%) and 55% (95% CI 39–71%), respectively.

Overall burnout was lower in trials using *adapted* MBI versions than in studies using MBI and other instruments. Furthermore, when the *adapted* MBI version was utilized to measure EE and DP, a lower prevalence was found than when the MBI versions were employed (Table 2).

**Table 2:** The results of the subgroup analysis for burnout and its three dimensions.

**Variable**	**Burnout**			**Emotional exhaustion**			**Depersonalization**			**Lack of personal accomplishment**		
	**N of study**	**Prevalence (95% CI)**	**I^**2**^**	**N of study**	**Prevalence (95% CI)**	**I^**2**^**	**N of study**	**Prevalence (95% CI)**	**I^**2**^**	**N of study**	**Prevalence (95% CI)**	**I^**2**^**
**Participants**												
Physicians and/or nurses	6	66 (51–81)	98.60	11	48 (34–62)	99.61	11	51 (33–70)	99.84	10	27 (23–31)	99.72
Mixed healthcare workers	7	40 (25–55)	99.29	8	56 (48–64)	95.80	8	53 (37–69)	99.02	8	29 (18–40)	98.41
**Frontline exposure** ^**I**^												
Yes	4	39 (21–57)	97.55	10	52 (42–63)	98.57	10	57 (35–78)	99.80	10	29 (17–41)	99.66
No	-	-	-	2	68 (64–71)	0.00	2	48 (44–52)	0.00	2	24 (20–27)	0.00
Mixed	7	55 (40–69)	99.29	5	51 (28–74)	99.75	5	47 (39–55)	96.19	5	26 (4–48)	99.70
**Time from beginning of pandemic**												
From beginning to 3 months	2	66 (64–68)	0.00	3	30 (-9–70)	0.00	3	34 (14–53)	0.00	3	48 (34–62)	0.00
3 months and afterwards	10	52 (39–66)	99.22	15	54 (46–62)	98.74	15	55 (39–71)	99.79	14	23 (21–26)	99.30
**Diagnostic tool for assessment of burnout**												
MBI	4	53 (29–76)	98.93	14	54 (46–63)	98.33	14	57 (40–74)	99.79	14	25 (23–28)	99.27
MBI (adapted version)	4	48 (28–69)	99.17	5	43 (17–70)	99.80	5	38 (27–48)	98.43	4	31 (10–53)	99.70
Other tools	5	54 (33–75)	99.49	-	-	-	-	-	-	-	-	
**Country income level**												
High income	7	56 (38–74)	99.42	9	55 (41–69)	98.65	9	46 (34–58)	98.28	8	32 (19–44)	98.44
Upper middle income	4	48 (28–69)	99.17	7	49 (32–65)	99.69	7	55 (30–79)	99.90	7	25 (21–30)	99.77
Lower middle income	-	-	-	2	29 (26–33)	0.00	2	64 (60–68)	0.00	2	12 (9–14)	0.00
**Sample size**												
<500	5	41 (15–66)	99.10	11	49 (34–64)	98.52	11	53 (34–71)	99.15	10	31 (21–42)	97.33
>500	7	59 (47–71)	99.20	7	51 (36–67)	99.73	7	44 (38–51)	98.18	7	29 (14–45)	99.82

*CI, confidence interval*.

*^I^Based on the exposure of the participant with COVID-19 patients in workplace, there were three categorizations: yes (only participants with exposure to COVID-19 cases were studied), no (participants did not exposed to COVID-19 patients) and mixed (both groups were studies)*.

In a subgroup examination of national income levels, overall burnout, EE, and lack of PA show a falling gradient from high, upper-middle, to lower middle-income countries. In the case of DP, however, the gradient is inverted.

Overall burnout was shown to be higher in studies with a sample size of more than 500 people at 59% (95% CI 47–71%).

## Discussion

### Summary of the Main Findings

This study aimed to present a comprehensive picture of the prevalence of burnout and its dimensions among various healthcare workers during the COVID-19 pandemic. Overall, the prevalence of burnout was 52% among all healthcare workers, with nurses and/or physicians experiencing the highest levels (66%), which is higher than rates reported in other studies performed during the past two decades [i.e., 32 to 34%] (60, 61). In addition, studies performed during the COVID-19 pandemic reported a rate of 37.4% (62). Hence, a more comprehensive assessment of burnout among healthcare workers during the COVID-19 pandemic is provided in this meta-analysis. The prevalence of burnout dimensions among healthcare workers, including EE, DP, and a lack of PA, was 51, 52, and 28%, respectively. In comparison to studies that measured burnout dimensions by means of MBI versions, studies that reported overall burnout used a variety of instruments. Hence, caution should be taken when interpreting the overall prevalence of burnout.

To the best of our knowledge, this is one of the first reviews to demonstrate that all healthcare workers, including physicians and nurses, may suffer from significant levels of overall burnout, EE, DP, and lack of PA. The highest prevalence of EE, DP, and lack of PA was found in studies that investigated a wide range of healthcare workers (56, 53, and 29%, respectively). Galanis et al. reviewed six studies on burnout among nurses during the COVID-19 pandemic and reported a pooled prevalence of 34.1, 12.6, and 15.2% for EE, DP, and PA, respectively (29), which the reported values are lower than that of the present study for nurses and/or physicians (48, 51, and 27%). The observed difference can be attributed to the larger number of reviewed articles in the present meta-analysis and the fact that our pooled prevalence includes burnout dimensions of physicians as well.

Many studies have focused on burnout among frontline healthcare workers due to higher risk and difficulties related to the management of COVID-19 patients (26). However, our findings revealed the considerable prevalence of EE and DP among non-frontline healthcare providers. Workplace stress, time constraints, and anxiety are all linked to burnout, which may explain the high rate of burnout among healthcare professionals who are not on the frontlines of the COVID-19 treatment (63). According to our meta-analysis, EE was more prevalent in non-frontlines, while frontlines had higher DP and lack of PA prevalence. Further studies are needed to extend our knowledge regarding the higher prevalence of EE in non-frontlines because we could identify only two related studies in non-frontlines (36, 44).

The prevalences of PA and DP were relatively similar in previous studies, both for studies performed in early pandemic and non-pandemic situations (29, 64, 65). Nevertheless, this meta-analysis showed that lack of PA (28%) was the least prevalent among the three burnout dimensions. The COVID-19 pandemic raised the workload of healthcare providers, which in turn led to a higher occurrence of EE and DP. However, it seems that lack of PA is not as prevalent as it is expected to be (66). A lower perceived lack of PA despite higher workload levels, can be attributed to the fact that healthcare worker feels more usefulness, altruism, appreciation by the community, and work meaningfulness during this pandemic, which might cause increased PA (67–69).

There are evidence indicating that healthcare providers experience a worsening rate or ascending slope of burnout over time (70, 71). Since more detrimental factors play a role in the epidemic condition, it can be argued that healthcare workers experience more burnout in this era over time. However, according to our subcategory analysis on the time of data collection, burnout has not increased from the first 3 months onwards. However, more follow-up studies are suggested.

Among the various valid tools used to assess burnout in included articles of this meta-analysis, MBI was the main one. Other infrequently used instruments included adaptive versions of MBI, the Copenhagen Burnout Inventory, the Oldenburg Burnout Inventory, and the Professional Fulfillment index. Despite this diversity, there is a similarity in burnout prevalence among a full version of MBI and other tools. Researchers might use the MBI full version or other instruments while considering the slightly different dimensions of burnout. However, this was not the case in adaptive versions of MBI. In studies in which adapted MBI versions were used, overall burnout prevalence was lower than MBI and other tools. This difference in burnout prevalence identified by adapted MBI versions vs. other instruments might partly be due to differences in applied methodologies, study participants, or the tool itself. This issue should be considered when interpreting the results obtained using adapted or modified versions of MBI.

Our findings indicated a gradient in the prevalence of burnout subcategories among HICs, UMICs, and LMICs. HICs had higher overall burnout, EE, and lack of PA than in UMICs and LMICs, respectively. In contrast, the prevalence of DP was higher in LMICs than UMICs and HICs. This discrepancy can be attributed to the higher COVID-19 burden in HICs early in the pandemic or publishing more studies on this issue in high or middle-income countries (18, 72). It is noteworthy to mention that factors associated with burnout in LMICs are different from those of HICs. Also, more experience working in conditions of high adversity and limited availability of supplies would lead to more resilience, and ultimately, less burnout in healthcare providers of LMICs (18).

Almost all included publications had good quality scores. However, we suggest paying more attention to reporting the source of funding and addressing potential bias sources in future studies.

Since the future of the COVID-19 pandemic is unpredictable, the epidemic situation has been prolonged and healthcare workers face brutal working conditions; health policymakers should pay a special emphasis on the mental health of healthcare staff. The prevalence of burnout, as an undesirable outcome of the pandemic, among healthcare workers has considerably increased during the COVID-19 pandemic. According to the findings, the authors recommend performing further studies on the prevalence of burnout, especially in LIC, factors associated with burnout, and cost-effective interventions that can effectively prevent and improve burnout. It is critical to consider interventions that can mitigate burnout during pandemics and develop psychological support for healthcare professionals that will protect not just the frontline from burnout, but also all the healthcare providers.

### Limitations

High heterogeneity of included studies is expected in the meta-analysis of prevalence studies. Excluding the articles that did not report the prevalence of burnout or its dimensions, which probably has affected the findings. We suggest developing a universal cut-off for assessing the prevalence of burnout in studies that only utilize the mean score of burnout.

## Conclusion

This study demonstrated that nearly half of the healthcare workers experienced burnout during the COVID-19 pandemic. Non-frontline COVID-19-exposed healthcare workers might experience burnout. Further studies on burnout in low and lower-middle-income countries are suggested. A uniform diagnostic tool for the assessment of burnout is warranted.

## Data Availability Statement

The original contributions presented in the study are included in the article/Supplementary Material, further inquiries can be directed to the corresponding author.

## Author Contributions

SG and KL initiated, conceptualized, and designed the study. SG, KL, and KH collaborated in analysis. SG, SA, and MY collaborated in data collection and processing. SG, SA, and KH wrote the manuscript. All the authors contributed in the edit of the manuscript and all critically reviewed manuscript.

## Conflict of Interest

The authors declare that the research was conducted in the absence of any commercial or financial relationships that could be construed as a potential conflict of interest.

## Publisher's Note

All claims expressed in this article are solely those of the authors and do not necessarily represent those of their affiliated organizations, or those of the publisher, the editors and the reviewers. Any product that may be evaluated in this article, or claim that may be made by its manufacturer, is not guaranteed or endorsed by the publisher.
