# Supplementary material for: A Systematic Review and Meta-Analysis of Burnout Among Healthcare Workers During COVID-19
Source: Front Psychiatry. 2021 Nov 10;12:758849. doi: 10.3389/fpsyt.2021.758849 (PMC8631719; doi:10.3389/fpsyt.2021.758849)
Supplement: Supplementary file 1 [file Table_1.docx]

**Supplementary table 1:** The search strings that were used for the four main databases.

| **PubMed**  ((Physician[tiab] OR Physicians[tiab] OR "Medical doctor"[tiab] OR MD[tiab] OR Doctor[tiab] OR Specialists[tiab] OR Specialist[tiab] OR Specialism[tiab] OR Specialist[tiab] OR "General Practitioners"[tiab] OR "General Practitioner"[tiab] OR GP[tiab] OR (Practitioner[tiab] AND General[tiab]) OR (Practitioners[tiab] AND General[tiab]) OR (Physicians[tiab] AND "General Practice"[tiab]) OR "General Practice Physician"[tiab] OR "General Practice Physicians"[tiab] OR (Physician[tiab] AND "General Practice"[tiab]) OR ("Practice Physicians"[tiab] AND General[tiab]) or "Health Staff"[tiab] OR (Personnel[tiab] AND Health[tiab]) OR "Health Care Providers"[tiab] OR "Health Care Provider"[tiab] OR (Provider[tiab] AND "Health Care"[tiab]) OR "Healthcare Providers"[tiab] OR "Healthcare Provider"[tiab] OR (Provider[tiab] AND Healthcare[tiab]) OR "Healthcare Workers"[tiab] OR "Healthcare Worker"[tiab] OR "Health Care Professionals"[tiab] OR "Health Care Professional"[tiab] OR (Professional[tiab] AND "Health Care"[tiab]) OR Allergists[tiab] OR Anesthesiologists[tiab] OR Cardiologists[tiab] OR Dermatologists[tiab] OR Endocrinologists[tiab] OR "Foreign Medical Graduates"[tiab] OR Gastroenterologists[tiab] OR Geriatricians[tiab] OR Hospitalists[tiab] OR Nephrologists[tiab] OR Neurologists[tiab] OR "Occupational Health Physicians"[tiab] OR Oncologists[tiab] OR "Radiation Oncologists"[tiab] OR Ophthalmologists[tiab] OR "Osteopathic Physicians"[tiab] OR Otolaryngologists[tiab] OR Pathologists[tiab] OR Pediatricians[tiab] OR Neonatologists[tiab] OR Physiatrists[tiab] OR (Physicians[tiab] AND Family[tiab]) OR (Physicians[tiab] AND "Primary Care"[tiab]) OR (Physicians[tiab] AND Women[tiab]) OR Pulmonologists[tiab] OR Radiologists[tiab] OR "Radiation Oncologists"[tiab] OR Rheumatologists[tiab] OR Surgeons[tiab] OR "Barber Surgeons"[tiab] OR Neurosurgeons[tiab] OR "Orthopedic Surgeons"[tiab] OR Urologists[tiab] OR "Laboratory Personnel"[tiab] OR Nurse[tiab] OR (Personnel[tiab] AND Nursing[tiab]) OR "Nursing Personnel"[tiab] OR "Registered Nurses"[tiab] OR (Nurse[tiab] AND Registered[tiab]) OR (Nurses[tiab] AND Registered[tiab]) OR "Registered Nurse"[tiab] OR "Health Personnel"[tiab] OR "Health worker"[tiab] OR "Health workers"[tiab] OR "Health caregivers"[tiab]) AND (COVID-19[tiab] OR "COVID 19"[tiab] OR "COVID-19 Virus Disease"[tiab] OR "COVID 19 Virus Disease"[tiab] OR "COVID-19 Virus Diseases"[tiab] OR (Disease[tiab] AND "COVID-19 Virus"[tiab]) OR ("Virus Disease"[tiab] AND COVID-19[tiab]) OR "COVID-19 Virus Infection"[tiab] OR "COVID 19 Virus Infection"[tiab] OR "COVID-19 Virus Infections"[tiab] OR "Infection[tiab] AND "COVID-19 Virus"[tiab]) OR ("Virus Infection"[tiab] AND COVID-19[tiab]) OR "2019-nCoV Infection"[tiab] OR "2019 nCoV Infection"[tiab] OR "2019-nCoV Infections"[tiab] OR (Infection[tiab] AND 2019-nCoV[tiab]) OR "Coronavirus Disease-19"[tiab] OR "Coronavirus Disease 19"[tiab] OR "2019 Novel Coronavirus Disease"[tiab] OR "2019 Novel Coronavirus Infection"[tiab] OR "2019-nCoV Disease"[tiab] OR "2019 nCoV Disease"[tiab] OR "2019-nCoV Diseases"[tiab] OR (Disease[tiab] AND 2019-nCoV[tiab]) OR COVID19[tiab] OR "Coronavirus Disease 2019"[tiab] OR ("Disease 2019"[tiab] AND Coronavirus[tiab]) OR "SARS Coronavirus 2 Infection"[tiab] OR "SARS-CoV-2 Infection"[tiab] OR (Infection[tiab] AND SARS-CoV-2[tiab]) OR "SARS CoV 2 Infection"[tiab] OR "SARS-CoV-2 Infections"[tiab] OR "COVID-19 Pandemic"[tiab] OR "COVID 19 Pandemic"[tiab] OR "COVID-19 Pandemics"[tiab] OR (Pandemic[tiab] AND COVID-19[tiab])) AND ("Psychological Burnout"[tiab] OR "Burn-out Syndrome"[tiab] OR "Burn out Syndrome"[tiab] OR Burnout[tiab] OR "Burnout Syndrome"[tiab] OR Burn-out[tiab] OR "Burn out"[tiab] OR "Psychological Burn-out"[tiab] OR (Burn-out[tiab] AND Psychological[tiab]) OR "Psychological Burn out"[tiab] OR "Professional Burnout"[tiab] OR "Occupational Burnout"[tiab] OR (Burnout[tiab] AND Occupational[tiab]) OR "Career Burnout"[tiab] OR (Burnout[tiab] AND Career[tiab])))  **N: 490** |
| --- |
| **Scopus**  ((TITLE-ABS(Physician) OR TITLE-ABS(Physicians) OR TITLE-ABS("Medical doctor") OR TITLE-ABS(MD) OR TITLE-ABS(Doctor) OR TITLE-ABS(Specialists) OR TITLE-ABS(Specialist) OR TITLE-ABS(Specialism) OR TITLE-ABS(Specialist) OR TITLE-ABS("General Practitioners") OR TITLE-ABS("General Practitioner") OR TITLE-ABS(GP) OR (TITLE-ABS(Practitioner) AND TITLE-ABS(General)) OR (TITLE-ABS(Practitioners) AND TITLE-ABS(General)) OR (TITLE-ABS(Physicians) AND TITLE-ABS("General Practice")) OR TITLE-ABS("General Practice Physician") OR TITLE-ABS("General Practice Physicians") OR (TITLE-ABS(Physician) AND TITLE-ABS("General Practice")) OR (TITLE-ABS("Practice Physicians") AND TITLE-ABS(General)) or TITLE-ABS("Health Staff") OR (TITLE-ABS(Personnel) AND TITLE-ABS(Health)) OR TITLE-ABS("Health Care Providers") OR TITLE-ABS("Health Care Provider") OR (TITLE-ABS(Provider) AND TITLE-ABS("Health Care")) OR TITLE-ABS("Healthcare Providers") OR TITLE-ABS("Healthcare Provider") OR (TITLE-ABS(Provider) AND TITLE-ABS(Healthcare)) OR TITLE-ABS("Healthcare Workers") OR TITLE-ABS("Healthcare Worker") OR TITLE-ABS("Health Care Professionals") OR TITLE-ABS("Health Care Professional") OR (TITLE-ABS(Professional) AND TITLE-ABS("Health Care")) OR TITLE-ABS(Allergists) OR TITLE-ABS(Anesthesiologists) OR TITLE-ABS(Cardiologists) OR TITLE-ABS(Dermatologists) OR TITLE-ABS(Endocrinologists) OR TITLE-ABS("Foreign Medical Graduates") OR TITLE-ABS(Gastroenterologists) OR TITLE-ABS(Geriatricians) OR TITLE-ABS(Hospitalists) OR TITLE-ABS(Nephrologists) OR TITLE-ABS(Neurologists) OR TITLE-ABS("Occupational Health Physicians") OR TITLE-ABS(Oncologists) OR TITLE-ABS("Radiation Oncologists") OR TITLE-ABS(Ophthalmologists) OR TITLE-ABS("Osteopathic Physicians") OR TITLE-ABS(Otolaryngologists) OR TITLE-ABS(Pathologists) OR TITLE-ABS(Pediatricians) OR TITLE-ABS(Neonatologists) OR TITLE-ABS(Physiatrists) OR (TITLE-ABS(Physicians) AND TITLE-ABS(Family)) OR (TITLE-ABS(Physicians) AND TITLE-ABS("Primary Care")) OR (TITLE-ABS(Physicians) AND TITLE-ABS(Women)) OR TITLE-ABS(Pulmonologists) OR TITLE-ABS(Radiologists) OR TITLE-ABS("Radiation Oncologists") OR TITLE-ABS(Rheumatologists) OR TITLE-ABS(Surgeons) OR TITLE-ABS("Barber Surgeons") OR TITLE-ABS(Neurosurgeons) OR TITLE-ABS("Orthopedic Surgeons") OR TITLE-ABS(Urologists) OR TITLE-ABS("Laboratory Personnel") OR TITLE-ABS(Nurse) OR (TITLE-ABS(Personnel) AND TITLE-ABS(Nursing)) OR TITLE-ABS("Nursing Personnel") OR TITLE-ABS("Registered Nurses") OR (TITLE-ABS(Nurse) AND TITLE-ABS(Registered)) OR (TITLE-ABS(Nurses) AND TITLE-ABS(Registered)) OR TITLE-ABS("Registered Nurse") OR TITLE-ABS("Health Personnel") OR TITLE-ABS("Health worker") OR TITLE-ABS("Health workers") OR TITLE-ABS("Health caregivers")) AND (TITLE-ABS(COVID-19) OR TITLE-ABS("COVID 19") OR TITLE-ABS("COVID-19 Virus Disease") OR TITLE-ABS("COVID 19 Virus Disease") OR TITLE-ABS("COVID-19 Virus Diseases") OR (TITLE-ABS(Disease) AND TITLE-ABS("COVID-19 Virus")) OR (TITLE-ABS("Virus Disease") AND TITLE-ABS(COVID-19)) OR TITLE-ABS("COVID-19 Virus Infection") OR TITLE-ABS("COVID 19 Virus Infection") OR TITLE-ABS("COVID-19 Virus Infections") OR (TITLE-ABS(Infection) AND TITLE-ABS("COVID-19 Virus")) OR (TITLE-ABS("Virus Infection") AND TITLE-ABS(COVID-19)) OR TITLE-ABS("2019-nCoV Infection") OR TITLE-ABS("2019 nCoV Infection") OR TITLE-ABS("2019-nCoV Infections") OR (TITLE-ABS(Infection) AND TITLE-ABS(2019-nCoV)) OR TITLE-ABS("Coronavirus Disease-19") OR TITLE-ABS("Coronavirus Disease 19") OR TITLE-ABS("2019 Novel Coronavirus Disease") OR TITLE-ABS("2019 Novel Coronavirus Infection") OR TITLE-ABS("2019-nCoV Disease") OR TITLE-ABS("2019 nCoV Disease") OR TITLE-ABS("2019-nCoV Diseases") OR (TITLE-ABS(Disease) AND TITLE-ABS(2019-nCoV)) OR TITLE-ABS(COVID19) OR TITLE-ABS("Coronavirus Disease 2019") OR (TITLE-ABS("Disease 2019") AND TITLE-ABS(Coronavirus)) OR TITLE-ABS("SARS Coronavirus 2 Infection") OR TITLE-ABS("SARS-CoV-2 Infection") OR (TITLE-ABS(Infection) AND TITLE-ABS(SARS-CoV-2)) OR TITLE-ABS("SARS CoV 2 Infection") OR TITLE-ABS("SARS-CoV-2 Infections") OR TITLE-ABS("COVID-19 Pandemic") OR TITLE-ABS("COVID 19 Pandemic") OR TITLE-ABS("COVID-19 Pandemics") OR (TITLE-ABS(Pandemic) AND TITLE-ABS(COVID-19))) AND (TITLE-ABS("Psychological Burnout") OR TITLE-ABS("Burn-out Syndrome") OR TITLE-ABS("Burn out Syndrome") OR TITLE-ABS(Burnout) OR TITLE-ABS("Burnout Syndrome") OR TITLE-ABS(Burn-out) OR TITLE-ABS("Burn out") OR TITLE-ABS("Psychological Burn-out") OR (TITLE-ABS(Burn-out) AND TITLE-ABS(Psychological)) OR TITLE-ABS("Psychological Burn out") OR TITLE-ABS("Professional Burnout") OR TITLE-ABS("Occupational Burnout") OR (TITLE-ABS(Burnout) AND TITLE-ABS(Occupational)) OR TITLE-ABS("Career Burnout") OR (TITLE-ABS(Burnout) AND TITLE-ABS(Career))))  **N: 189** |
| **WoS**  ((TS=(Physician) OR TS=(Physicians) OR TS=("Medical doctor") OR TS=(MD) OR TS=(Doctor) OR TS=(Specialists) OR TS=(Specialist) OR TS=(Specialism) OR TS=(Specialist) OR TS=("General Practitioners") OR TS=("General Practitioner") OR TS=(GP) OR (TS=(Practitioner) AND TS=(General)) OR (TS=(Practitioners) AND TS=(General)) OR (TS=(Physicians) AND TS=("General Practice")) OR TS=("General Practice Physician") OR TS=("General Practice Physicians") OR (TS=(Physician) AND TS=("General Practice")) OR (TS=("Practice Physicians") AND TS=(General)) or TS=("Health Staff") OR (TS=(Personnel) AND TS=(Health)) OR TS=("Health Care Providers") OR TS=("Health Care Provider") OR (TS=(Provider) AND TS=("Health Care")) OR TS=("Healthcare Providers") OR TS=("Healthcare Provider") OR (TS=(Provider) AND TS=(Healthcare)) OR TS=("Healthcare Workers") OR TS=("Healthcare Worker") OR TS=("Health Care Professionals") OR TS=("Health Care Professional") OR (TS=(Professional) AND TS=("Health Care")) OR TS=(Allergists) OR TS=(Anesthesiologists) OR TS=(Cardiologists) OR TS=(Dermatologists) OR TS=(Endocrinologists) OR TS=("Foreign Medical Graduates") OR TS=(Gastroenterologists) OR TS=(Geriatricians) OR TS=(Hospitalists) OR TS=(Nephrologists) OR TS=(Neurologists) OR TS=("Occupational Health Physicians") OR TS=(Oncologists) OR TS=("Radiation Oncologists") OR TS=(Ophthalmologists) OR TS=("Osteopathic Physicians") OR TS=(Otolaryngologists) OR TS=(Pathologists) OR TS=(Pediatricians) OR TS=(Neonatologists) OR TS=(Physiatrists) OR (TS=(Physicians) AND TS=(Family)) OR (TS=(Physicians) AND TS=("Primary Care")) OR (TS=(Physicians) AND TS=(Women)) OR TS=(Pulmonologists) OR TS=(Radiologists) OR TS=("Radiation Oncologists") OR TS=(Rheumatologists) OR TS=(Surgeons) OR TS=("Barber Surgeons") OR TS=(Neurosurgeons) OR TS=("Orthopedic Surgeons") OR TS=(Urologists) OR TS=("Laboratory Personnel") OR TS=(Nurse) OR (TS=(Personnel) AND TS=(Nursing)) OR TS=("Nursing Personnel") OR TS=("Registered Nurses") OR (TS=(Nurse) AND TS=(Registered)) OR (TS=(Nurses) AND TS=(Registered)) OR TS=("Registered Nurse") OR TS=("Health Personnel") OR TS=("Health worker") OR TS=("Health workers") OR TS=("Health caregivers")) AND (TS=(COVID-19) OR TS=("COVID 19") OR TS=("COVID-19 Virus Disease") OR TS=("COVID 19 Virus Disease") OR TS=("COVID-19 Virus Diseases") OR (TS=(Disease) AND TS=("COVID-19 Virus")) OR (TS=("Virus Disease") AND TS=(COVID-19)) OR TS=("COVID-19 Virus Infection") OR TS=("COVID 19 Virus Infection") OR TS=("COVID-19 Virus Infections") OR (TS=(Infection) AND TS=("COVID-19 Virus")) OR (TS=("Virus Infection") AND TS=(COVID-19)) OR TS=("2019-nCoV Infection") OR TS=("2019 nCoV Infection") OR TS=("2019-nCoV Infections") OR (TS=(Infection) AND TS=(2019-nCoV)) OR TS=("Coronavirus Disease-19") OR TS=("Coronavirus Disease 19") OR TS=("2019 Novel Coronavirus Disease") OR TS=("2019 Novel Coronavirus Infection") OR TS=("2019-nCoV Disease") OR TS=("2019 nCoV Disease") OR TS=("2019-nCoV Diseases") OR (TS=(Disease) AND TS=(2019-nCoV)) OR TS=(COVID19) OR TS=("Coronavirus Disease 2019") OR (TS=("Disease 2019") AND TS=(Coronavirus)) OR TS=("SARS Coronavirus 2 Infection") OR TS=("SARS-CoV-2 Infection") OR (TS=(Infection) AND TS=(SARS-CoV-2)) OR TS=("SARS CoV 2 Infection") OR TS=("SARS-CoV-2 Infections") OR TS=("COVID-19 Pandemic") OR TS=("COVID 19 Pandemic") OR TS=("COVID-19 Pandemics") OR (TS=(Pandemic) AND TS=(COVID-19))) AND (TS=("Psychological Burnout") OR TS=("Burn-out Syndrome") OR TS=("Burn out Syndrome") OR TS=(Burnout) OR TS=("Burnout Syndrome") OR TS=(Burn-out) OR TS=("Burn out") OR TS=("Psychological Burn-out") OR (TS=(Burn-out) AND TS=(Psychological)) OR TS=("Psychological Burn out") OR TS=("Professional Burnout") OR TS=("Occupational Burnout") OR (TS=(Burnout) AND TS=(Occupational)) OR TS=("Career Burnout") OR (TS=(Burnout) AND TS=(Career))))  **N: 172** |
| Embase  ((Physician:ti,ab OR Physicians:ti,ab OR "Medical doctor":ti,ab OR MD:ti,ab OR Doctor:ti,ab OR Specialists:ti,ab OR Specialist:ti,ab OR Specialism:ti,ab OR Specialist:ti,ab OR "General Practitioners":ti,ab OR "General Practitioner":ti,ab OR GP:ti,ab OR (Practitioner:ti,ab AND General:ti,ab) OR (Practitioners:ti,ab AND General:ti,ab) OR (Physicians:ti,ab AND "General Practice":ti,ab) OR "General Practice Physician":ti,ab OR "General Practice Physicians":ti,ab OR (Physician:ti,ab AND "General Practice":ti,ab) OR ("Practice Physicians":ti,ab AND General:ti,ab) or "Health Staff":ti,ab OR (Personnel:ti,ab AND Health:ti,ab) OR "Health Care Providers":ti,ab OR "Health Care Provider":ti,ab OR (Provider:ti,ab AND "Health Care":ti,ab) OR "Healthcare Providers":ti,ab OR "Healthcare Provider":ti,ab OR (Provider:ti,ab AND Healthcare:ti,ab) OR "Healthcare Workers":ti,ab OR "Healthcare Worker":ti,ab OR "Health Care Professionals":ti,ab OR "Health Care Professional":ti,ab OR (Professional:ti,ab AND "Health Care":ti,ab) OR Allergists:ti,ab OR Anesthesiologists:ti,ab OR Cardiologists:ti,ab OR Dermatologists:ti,ab OR Endocrinologists:ti,ab OR "Foreign Medical Graduates":ti,ab OR Gastroenterologists:ti,ab OR Geriatricians:ti,ab OR Hospitalists:ti,ab OR Nephrologists:ti,ab OR Neurologists:ti,ab OR "Occupational Health Physicians":ti,ab OR Oncologists:ti,ab OR "Radiation Oncologists":ti,ab OR Ophthalmologists:ti,ab OR "Osteopathic Physicians":ti,ab OR Otolaryngologists:ti,ab OR Pathologists:ti,ab OR Pediatricians:ti,ab OR Neonatologists:ti,ab OR Physiatrists:ti,ab OR (Physicians:ti,ab AND Family:ti,ab) OR (Physicians:ti,ab AND "Primary Care":ti,ab) OR (Physicians:ti,ab AND Women:ti,ab) OR Pulmonologists:ti,ab OR Radiologists:ti,ab OR "Radiation Oncologists":ti,ab OR Rheumatologists:ti,ab OR Surgeons:ti,ab OR "Barber Surgeons":ti,ab OR Neurosurgeons:ti,ab OR "Orthopedic Surgeons":ti,ab OR Urologists:ti,ab OR "Laboratory Personnel":ti,ab OR Nurse:ti,ab OR (Personnel:ti,ab AND Nursing:ti,ab) OR "Nursing Personnel":ti,ab OR "Registered Nurses":ti,ab OR (Nurse:ti,ab AND Registered:ti,ab) OR (Nurses:ti,ab AND Registered:ti,ab) OR "Registered Nurse":ti,ab OR "Health Personnel":ti,ab OR "Health worker":ti,ab OR "Health workers":ti,ab OR "Health caregivers":ti,ab) AND (COVID-19:ti,ab OR "COVID 19":ti,ab OR "COVID-19 Virus Disease":ti,ab OR "COVID 19 Virus Disease":ti,ab OR "COVID-19 Virus Diseases":ti,ab OR (Disease:ti,ab AND "COVID-19 Virus":ti,ab) OR ("Virus Disease":ti,ab AND COVID-19:ti,ab) OR "COVID-19 Virus Infection":ti,ab OR "COVID 19 Virus Infection":ti,ab OR "COVID-19 Virus Infections":ti,ab OR (Infection:ti,ab AND "COVID-19 Virus":ti,ab) OR ("Virus Infection":ti,ab AND COVID-19:ti,ab) OR "2019-nCoV Infection":ti,ab OR "2019 nCoV Infection":ti,ab OR "2019-nCoV Infections":ti,ab OR (Infection:ti,ab AND 2019-nCoV:ti,ab) OR "Coronavirus Disease-19":ti,ab OR "Coronavirus Disease 19":ti,ab OR "2019 Novel Coronavirus Disease":ti,ab OR "2019 Novel Coronavirus Infection":ti,ab OR "2019-nCoV Disease":ti,ab OR "2019 nCoV Disease":ti,ab OR "2019-nCoV Diseases":ti,ab OR (Disease:ti,ab AND 2019-nCoV:ti,ab) OR COVID19:ti,ab OR "Coronavirus Disease 2019":ti,ab OR ("Disease 2019":ti,ab AND Coronavirus:ti,ab) OR "SARS Coronavirus 2 Infection":ti,ab OR "SARS-CoV-2 Infection":ti,ab OR (Infection:ti,ab AND SARS-CoV-2:ti,ab) OR "SARS CoV 2 Infection":ti,ab OR "SARS-CoV-2 Infections":ti,ab OR "COVID-19 Pandemic":ti,ab OR "COVID 19 Pandemic":ti,ab OR "COVID-19 Pandemics":ti,ab OR (Pandemic:ti,ab AND COVID-19:ti,ab)) AND ("Psychological Burnout":ti,ab OR "Burn-out Syndrome":ti,ab OR "Burn out Syndrome":ti,ab OR Burnout:ti,ab OR "Burnout Syndrome":ti,ab OR Burn-out:ti,ab OR "Burn out":ti,ab OR "Psychological Burn-out":ti,ab OR (Burn-out:ti,ab AND Psychological:ti,ab) OR "Psychological Burn out":ti,ab OR "Professional Burnout":ti,ab OR "Occupational Burnout":ti,ab OR (Burnout:ti,ab AND Occupational:ti,ab) OR "Career Burnout":ti,ab OR (Burnout:ti,ab AND Career:ti,ab)))  **N: 168** |
