# Supplementary material for: A Systematic Review and Meta-Analysis of Burnout Among Healthcare Workers During COVID-19
Source: Front Psychiatry. 2021 Nov 10;12:758849. doi: 10.3389/fpsyt.2021.758849 (PMC8631719; doi:10.3389/fpsyt.2021.758849)
Supplement: Supplementary file 2 [file Table_2.docx]

| Supplementary Table 2: the quality assessments of the twenty-seven original cross-sectional studies included in this study using the Strengthening the Reporting of Observational Studies in Epidemiology (STROBE) checklist | | | | | | | | | | | | | | | | | | | | | | | | | |  |
| --- | --- | --- | --- | --- | --- | --- | --- | --- | --- | --- | --- | --- | --- | --- | --- | --- | --- | --- | --- | --- | --- | --- | --- | --- | --- | --- |
| # | Item number of STROBE^*^ | 1 | 2 | 3 | 4 | 5 | 6 | 7 | 8 | 9 | 10 | 11 | 12 | 13 | 14 | 15 | 16 | 17 | 18 | 19 | 20 | 21 | 22 | Total | Quality |  |
|  | Author year |  |  |  |  |  |  |  |  |  |  |  |  |  |  |  |  |  |  |  |  |  |  |  |  |  |
| 1 | Abdelghani(2020) | 2 | 2 | 2 | 2 | 2 | 2 | 2 | 2 | 2 | 2 | 2 | 2 | 2 | 2 | 2 | 2 | 2 | 2 | 2 | 2 | 2 | 2 | 44 | Good |  |
| 2 | Abdelhafiz(2020) | 2 | 2 | 2 | 2 | 2 | 2 | 2 | 2 | 1 | 2 | 2 | 2 | 2 | 2 | 2 | 2 | 2 | 2 | 2 | 2 | 2 | 0 | 41 | Good |  |
| 3 | Azoulay(2020) | 2 | 2 | 2 | 2 | 2 | 1 | 2 | 2 | 1 | 2 | 2 | 2 | 2 | 2 | 2 | 2 | 2 | 2 | 2 | 2 | 2 | 2 | 42 | Good |  |
| 4 | Chen, J (2020) | 2 | 2 | 2 | 2 | 2 | 1 | 2 | 2 | 1 | 2 | 2 | 2 | 2 | 2 | 2 | 2 | 2 | 2 | 2 | 2 | 2 | 2 | 42 | Good |  |
| 5 | Chen, R (2021) | 2 | 2 | 2 | 2 | 1 | 2 | 2 | 2 | 2 | 2 | 2 | 2 | 2 | 2 | 2 | 2 | 2 | 2 | 2 | 2 | 1 | 2 | 42 | Good |  |
| 6 | Di Monte(2020) | 2 | 2 | 2 | 2 | 2 | 2 | 2 | 2 | 2 | 0 | 2 | 2 | 2 | 2 | 2 | 2 | 2 | 2 | 2 | 2 | 2 | 0 | 40 | Good |  |
| 7 | Dobson (2021) | 1 | 2 | 2 | 2 | 2 | 0 | 2 | 2 | 2 | 2 | 2 | 2 | 2 | 2 | 2 | 2 | 2 | 2 | 2 | 2 | 1 | 2 | 40 | Good |  |
| 8 | Duarte(2020) | 2 | 2 | 1 | 2 | 2 | 2 | 2 | 2 | 1 | 2 | 2 | 2 | 2 | 2 | 2 | 2 | 2 | 2 | 2 | 2 | 2 | 2 | 42 | Good |  |
| 9 | Elhadi(2020) | 2 | 2 | 2 | 2 | 2 | 2 | 2 | 2 | 1 | 2 | 2 | 2 | 2 | 2 | 2 | 2 | 2 | 2 | 2 | 2 | 2 | 0 | 41 | Good |  |
| 10 | Evanoff(2020) | 2 | 2 | 2 | 2 | 2 | 2 | 2 | 2 | 1 | 2 | 2 | 2 | 2 | 2 | 2 | 2 | 2 | 2 | 2 | 2 | 2 | 2 | 43 | Good |  |
| 11 | Giusti(2020) | 2 | 2 | 2 | 2 | 2 | 2 | 2 | 2 | 2 | 2 | 2 | 2 | 2 | 2 | 2 | 2 | 2 | 2 | 2 | 2 | 2 | 0 | 42 | Good |  |
| 12 | Gomez(2020) | 2 | 2 | 2 | 2 | 2 | 2 | 2 | 2 | 2 | 2 | 2 | 2 | 2 | 1 | 2 | 2 | 2 | 2 | 1 | 2 | 2 | 0 | 40 | Good |  |
| 13 | Hu(2020) | 2 | 2 | 2 | 2 | 2 | 2 | 2 | 2 | 2 | 2 | 2 | 2 | 2 | 2 | 2 | 2 | 2 | 2 | 2 | 2 | 2 | 2 | 44 | Good |  |
| 14 | Kholmogorova(2020) | 2 | 2 | 2 | 2 | 2 | 2 | 1 | 2 | 2 | 2 | 1 | 0 | 0 | 2 | 2 | 2 | 2 | 2 | 2 | 1 | 2 | 1 | 36 | Fair |  |
| 15 | Lázaro-Pérez(2020) | 1 | 2 | 2 | 2 | 2 | 1 | 2 | 2 | 1 | 2 | 2 | 2 | 1 | 2 | 2 | 2 | 2 | 2 | 2 | 2 | 2 | 2 | 40 | Good |  |
| 16 | Liu(2020) | 2 | 2 | 2 | 2 | 1 | 1 | 2 | 2 | 2 | 2 | 2 | 2 | 1 | 2 | 2 | 2 | 2 | 2 | 2 | 2 | 2 | 2 | 41 | Good |  |
| 17 | Luceño-Moreno(2020) | 2 | 2 | 2 | 2 | 1 | 2 | 2 | 2 | 2 | 2 | 2 | 2 | 2 | 2 | 2 | 2 | 2 | 2 | 2 | 2 | 2 | 2 | 43 | Good |  |
| 18 | Martínez-López(2020) | 2 | 2 | 2 | 2 | 2 | 2 | 2 | 2 | 2 | 2 | 2 | 2 | 1 | 2 | 2 | 2 | 2 | 2 | 2 | 1 | 2 | 2 | 42 | Good |  |
| 19 | Miguel-Puga(2020) | 2 | 2 | 2 | 1 | 1 | 2 | 2 | 2 | 2 | 2 | 2 | 2 | 2 | 1 | 2 | 2 | 2 | 2 | 2 | 2 | 2 | 0 | 39 | Good |  |
| 20 | Park(2020) | 2 | 2 | 2 | 2 | 2 | 2 | 2 | 2 | 1 | 2 | 2 | 2 | 2 | 2 | 2 | 2 | 2 | 2 | 2 | 2 | 2 | 2 | 43 | Good |  |
| 21 | Ruiz-Fernández(2020) | 2 | 2 | 2 | 2 | 2 | 2 | 2 | 2 | 1 | 2 | 2 | 2 | 2 | 2 | 2 | 2 | 2 | 2 | 0 | 2 | 0 | 2 | 39 | Good |  |
| 22 | Sayilan(2020) | 1 | 2 | 2 | 2 | 2 | 2 | 2 | 2 | 2 | 2 | 2 | 2 | 2 | 2 | 2 | 2 | 2 | 2 | 2 | 2 | 0 | 0 | 39 | Good |  |
| 23 | Sayzni Roslan (2021) | 2 | 2 | 2 | 2 | 2 | 2 | 2 | 2 | 2 | 2 | 2 | 2 | 2 | 2 | 2 | 2 | 2 | 2 | 2 | 2 | 2 | 2 | 44 | Good |  |
| 24 | Tan(2020) | 2 | 2 | 2 | 2 | 2 | 2 | 2 | 2 | 2 | 2 | 2 | 2 | 2 | 2 | 2 | 2 | 2 | 2 | 2 | 2 | 2 | 2 | 44 | Good |  |
| 25 | W Khasne(2020) | 2 | 2 | 2 | 1 | 0 | 1 | 2 | 2 | 2 | 2 | 2 | 2 | 1 | 2 | 2 | 2 | 2 | 2 | 2 | 2 | 2 | 2 | 39 | Good |  |
| 26 | Wit(2020) | 2 | 2 | 2 | 2 | 2 | 2 | 2 | 2 | 2 | 2 | 2 | 2 | 2 | 2 | 2 | 2 | 2 | 2 | 2 | 2 | 2 | 2 | 44 | Good |  |
| 27 | Zhang(2020) | 2 | 2 | 2 | 2 | 2 | 2 | 2 | 2 | 1 | 2 | 2 | 2 | 2 | 2 | 2 | 2 | 2 | 2 | 2 | 2 | 2 | 0 | 41 | Good |  |
| *for all items,”1” represents for presence of item, “0“indicates lack of presence and “?” means criteria was not met completely or was not applicable.  For calculation of total study quality, “0”, “? “ And “1” were scored as 0, 1 and 2 respectively. Then sum of scores for assessment of each study’s quality was calculated. Each study was rated as either good (i.e., most criteria met with a low risk of bias, score 39-44), fair (i.e., some criteria met with a moderate risk of bias, score of 33-38), or poor (i.e., few criteria met and with a high risk of bias, score less than 33). | | | | | | | | | | | | | | | | | | | | | | | | | | |
